# Supplementary material for: Hybrid brain-computer interface using error-related potential and reinforcement learning
Source: Front Hum Neurosci. 2025 Jun 4;19:1569411. doi: 10.3389/fnhum.2025.1569411 (PMC12174104; doi:10.3389/fnhum.2025.1569411)
Supplement: Supplementary file 1 [file Data_Sheet_1.pdf]

Supplementary Material

1 NUMBER OF TRIALS IN BCI COMPETITION IV DATASET

Table S1. Total number of trials for each subject in the open-source dataset BCI Competition IV 2b used in this study (n = 9).

	B01	B02	B03	B04	B05	B06	B07	B08	B09	condition
training	200	200	200	210	210	200	200	220	200	left
	200	200	200	210	210	200	200	220	200	right
	**400**	**400**	**400**	**420**	**420**	**400**	**400**	**440**	**400**	**total**
evaluation	160	140	160	160	160	160	160	160	160	left
	160	140	160	160	160	160	160	160	160	right
	**320**	**280**	**320**	**320**	**320**	**320**	**320**	**320**	**320**	**total**

2 NUMBER OF TRIALS IN THE IN-HOUSE DATASET

Table S2. Total number of trials for each subject in the in-house dataset used in this study (n = 21).

S01	S02	S03	S05	S06	S07	S10	S11	S12	S13	S14	condition
356	247	395	388	249	293	241	343	350	315	307	left
334	240	387	401	256	285	253	356	338	322	317	right
**690**	**487**	**782**	**789**	**505**	**578**	**494**	**699**	**688**	**637**	**624**	**total**
S16	S17	S18	S19	S23	S24	S27	S28	S29	S30		condition
245	258	286	218	147	175	393	239	258	283		left
237	279	275	231	153	197	392	267	263	279		right
**482**	**537**	**561**	**449**	**300**	**372**	**785**	**506**	**521**	**562**		**total**

3 SCORES IN THE MOVEMENT IMAGERY QUESTIONNAIRE-3 (MIQ-3)

Table S3. Scores in the Movement Imagery Questionnaire-3 (MI3) for each subject recorded with our proposed protocol. Note that NA stands for not available and cover the subjects that participated in the pilot studies before we included the use of the questionnaire in the experimental protocol. Pilot subject $S07$ kindly returned to the lab and responded to the questionnaire after we introduce it to the protocol.

subject	internal imagery ability	external imagery ability	kinesthetic imagery ability	total MI ability
S01	NA	NA	NA	NA
S02	NA	NA	NA	NA
S03	NA	NA	NA	NA
S05	NA	NA	NA	NA
S06	NA	NA	NA	NA
S07	6.25	3.75	6	16
S10	NA	NA	NA	NA
S11	5.5	6	3.5	15
S12	4.75	5.25	3.25	13.25
S13	5.5	5.75	5.75	17
S14	5.25	5.5	4.25	15
S16	6.25	5.25	5.75	17.25
S17	6.26	3.75	6	16
S18	2.75	6	5	13.75
S19	6	5.5	6.25	17.75
S23	6.25	6.75	5.5	18.5
S24	5.75	5.75	6	17
S27	3.5	6.25	5	14.75
S28	6.25	5	6.25	17.5
S29	6	6.75	6.5	19.25
S30	6	6	5.5	17.5
**mean ± std (min-max)**	**5.47 ± 1.05 (2.75, 6.25)**	**5.58 ± 0.78 (4.75, 6.75)**	**5.37 ± 0.99 (3.25, 6.25)**	**16.37 ± 1.73 (13.25, 19.25)**
